# A Cross-Sectional Study Examining the Parametric Thyroid Feedback Quantile Index and Its Relationship with Metabolic and Cardiovascular Diseases

**DOI:** 10.1089/thy.2022.0025

**Published:** 2022-12-13

**Authors:** Vanesa Alonso-Ventura, Fernando Civeira, Almendra Alvarado-Rosas, Jose Manuel Lou-Bonafonte, Pilar Calmarza, Belen Moreno-Franco, Maria Jesus Andres-Otero, Fernando Calvo-Gracia, Patricia de Diego-Garcia, Martin Laclaustra

**Affiliations:** ^1^ Hospital Universitario Miguel Servet, IIS Aragón, Zaragoza, Spain.; ^2^ Instituto de Investigación Sanitaria de Aragón (IIS Aragón), Zaragoza, Spain.; ^3^ Facultad de Medicina, Universidad de Zaragoza, Spain.; ^4^ CIBERCV-ISCIII, Madrid, Spain.; ^5^ Instituto Agroalimentario de Aragón, CITA-Universidad de Zaragoza, Zaragoza, Spain.; ^6^ CIBEROBN-ISCIII, Madrid, Spain.; ^7^ Hospital Clínico Universitario Lozano Blesa, IIS Aragón, Zaragoza, Spain.

**Keywords:** atrial fibrillation, coronary heart disease, thyroid regulation, type 2 diabetes mellitus

## Abstract

**Background:**

The usual inverse correlation between thyrotropin (TSH) and thyroid hormone disappears in syndromes of central resistance to thyroid hormone, where both are high. TSH and thyroid hormone are also simultaneously high when there is an elevation of the set point of the thyroid regulation axis. This can be estimated with indices, such as the Parametric Thyroid Feedback Quantile-based Index (PTFQI), which was designed for the general population. The PTFQI is positively associated with diabetes prevalence, but association with other pathologies has not been yet explored. The aim of this project was to explore the potential relationship of the PTFQI with metabolic and cardiovascular disease in a sample of ambulatory adult patients from Spain.

**Methods:**

A cross-sectional study was carried out among the patients who underwent thyroid hormones measurement (6434 measurements from September to November 2018 in a central laboratory in Spain). We retrospectively reviewed clinical records of a subgroup of adults aged >18 years with normal TSH and free thyroxine (fT4) belonging to groups that represent extreme PTFQI (*n* = 661). Individuals with known conditions interfering the thyroid axis were excluded (remaining *n* = 296). Logistic and linear regression models adjusted for age and sex were used to calculate odds ratio (OR) of diseases and differences of clinical parameters, and 95% confidence intervals [CI].

**Results:**

Across levels with higher PTFQI, there was an increase in the prevalence of type 2 diabetes (High vs. Low PTFQI OR: 2.88 [CI: 1.14–7.86], *p*-Trend = 0.02), ischemic heart disease (16.4% vs. 0%, unadjusted Haldane–Anscombe corrected OR: 23.90 [CI: 1.36–21.48], adjusted *p*-Trend = 0.04), atrial fibrillation (OR: 8.13 [CI: 1.33–158.20], *p*-Trend = 0.05), and hypertension (OR: 3.19 [CI: 1.14–9.94], *p*-Trend = 0.05). While the prevalence of type 2 diabetes was similarly associated with TSH and fT4, ischemic heart disease, atrial fibrillation, and hypertension were more strongly associated with the differences in fT4 values.

**Conclusions:**

Type 2 diabetes, ischemic heart disease, atrial fibrillation, and hypertension may be associated with a higher central regulation set point for thyroid hormone. These findings should be confirmed in other populations.

## Introduction

Thyroid hormone has a critical role in energy balance: increases energy demands stimulating multiple synthetic and catabolic metabolic pathways, activates thermogenesis, intensifies the effect of the sympathetic nervous system, and increases food intake.^1^ The serum levels of thyrotropin (TSH) and thyroid hormone, both within their normal range, have been occasionally associated with energy-related diseases, mainly metabolic disorders,^2–6^ but also heart diseases.^7–9^ Interestingly, no association with ischemic heart disease has been demonstrated in euthyroid subjects.^7,8,10^ However, the interplay of the TSH–thyroid hormone has been seldom taken into account in these subjects.^3^

We have recently reported that a central (i.e., presumed pituitary) upregulation of thyroid hormone synthesis is associated with type 2 diabetes.^11^ We used an index specifically developed to quantify estimated pituitary inhibition by thyroid hormone in the general population, the Parametric Thyroid Feedback Quantile-based Index (PTFQI). The index quantifies the deviations from the physiological population-average pituitary response to thyroid hormone. It ranges from −1 to +1, with negative values representing an inappropriately low TSH for the actual thyroid hormone levels (a downregulated set point) and positive values, the opposite.

The aim of this work was to explore the potential relationship between an estimate of central thyroid regulation with metabolic and cardiovascular diseases in ambulatory adults. Our specific focus is on a subgroup of euthyroid patients with extreme PTFQI values.

## Materials and Methods

### Design and subjects

In this cross-sectional study, we screened all consecutive patients who underwent a thyroid hormone measurement between September and November 2018 (6434 analyses). The analyses were performed in the central laboratory of the Hospital Universitario “Miguel Servet” in Zaragoza (Spain), which is part of the national public health service and serves a population of 379,225 citizens. Analyses of patients older than 18 years (6051 analyses) were included to obtain the average values and distribution of thyroid hormone function for PTFQI calculation (as described below, in the “PTFQI and thyroid regulation space” subsection). Within these, having free thyroxine (fT4) and TSH within the normal range (5248 analyses) was the primary selection criteria. Among them, a sample was obtained (for retrospective clinical record review), belonging to one of five nonoverlapping groups (as described below, in the “Thyroid function regulation groups” subsection) across the thyroid regulation space (661 patients).

**FIG. 1.**
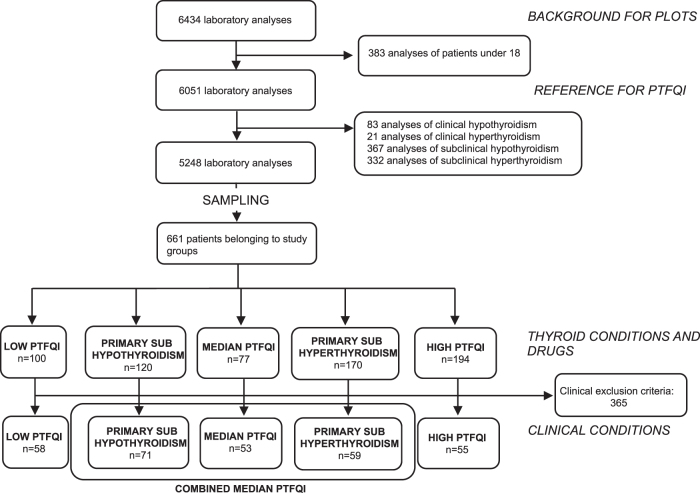
Flowchart. PTFQI, Parametric Thyroid Feedback Quantile-based Index.

Non-random ordered sampling was carried out to increase sensitivity for differences between PTFQI extremes while still being able to representatively describe a gradient across the space. Within reviewed records, a secondary selection (for the detailed analysis of group comparisons) excluded participants if they fulfilled one of the exclusion criteria, leaving 296 patients in the final analysis (Fig. 1). Some examples of the exclusion criteria included: pregnancy, known thyroid diseases and their treatment, interfering drugs and diseases, and advanced diseases (neoplasms, recent long hospital stays, malnutrition) (Supplementary Data). Most frequently found criteria appear in Table 1.

**Table 1.** Exclusion Criteria by Thyroid Regulation Groups

Exclusion criteria	*N* excluded	Low PTFQI	Combined median PTFQI			High PTFQI	*p*-Heter 5 groups	*p*-Trend 3 groups
			Primary subhypothyroidism	Median PTFQI	Primary subhyperthyroidism			
		% [*n*]	% [*n*]	% [*n*]	% [*n*]	% [*n*]		
	n/N	(*N* = 100)	(*N* = 120)	(*N* = 77)	(*N* = 170)	(*N* = 194)		
Overall	365/661	42.0 [42]	40.8 [49]	31.2 [24]	65.3 [111]	71.6 [139]	—	—
Pregnancy	14/661	13.0 [13]	0.0 [0]	1.3 [1]	0.0 [0]	0.0 [0]		
		1.00 (Ref.)	—	—	—	—	<0.01	<0.01
Treatment with levothyroxine	240/661	19.0 [19]	21.7 [26]	16.9 [13]	45.9 [78]	53.6 [104]		
		1.00 (Ref.)	1.21 [0.62–2.42]	0.96 [0.42–2.11]	4.64 [2.54–8.81]	6.43 [3.54–12.15]	<0.01	<0.01
Treatment with steroids	46/661	7.0 [7]	6.7 [8]	5.2 [4]	11.2 [19]	4.1 [8]		
		1.00 (Ref.)	0.88 [0.30–2.63]	0.63 [0.16–2.21]	1.39 [0.57–3.77]	0.47 [0.16–1.41]	0.12	0.09
Treatment with amiodarone	27/661	0.0 [0]	0.8 [1]	1.3 [1]	2.9 [5]	10.3 [20]		
		1.00 (Ref.)	—	—	—	—	<0.01	<0.01
Thyroidectomy for cancer	24/661	1.0 [1]	0.8 [1]	2.6 [2]	8.8 [15]	2.6 [5]		
		1.00 (Ref.)	0.86 [0.03–22.03]	2.78 [0.26–60.92]	10.38 [1.98–191.77]	2.84 [0.43–55.59]	<0.01	0.88
Treatment with radioactive iodine	22/661	4.0 [4]	2.5 [3]	2.6 [2]	2.9 [5]	4.1 [8]		
		1.00 (Ref.)	0.51 [0.10–2.42]	0.55 [0.07–2.95]	0.59 [0.15–2.52]	0.82 [0.24–3.28]	0.88	0.98
Pre-existent thyroid disorders	15/661	1.0 [1]	4.2[5]	0.0 [0]	4.1 [7]	1.0 [2]		
		1.00 (Ref.)	—	—	—	—	0.04	0.66
Type 1 diabetes mellitus	15/661	0.0 [0]	2.5 [3]	5.2 [4]	3.5 [6]	1.0 [2]		
		1.00 (Ref.)	—	—	—	—	0.03	0.68
Thyroidectomy for goiter	14/661	1.0 [1]	1.7 [2]	2.6 [2]	1.8 [3]	3.1 [6]		
		1.00 (Ref.)	1.49 [0.14–32.64]	2.60 [0.24–57.18]	1.76 [0.21–36.85]	3.08 [0.48–60.27]	0.77	0.23
Other exclusion criteria	25/661	5.0 [5]	4.2 [5]	1.3 [1]	4.7 [8]	3.1 [6]	—	—

Data are expressed in % [*n*]. OR [CI] from models adjusted for age and sex, referenced to the Low PTFQI group. *p*-Heter is the *p*-value for testing differences among groups. *p*-Trend is the *p*-value from a regression model that includes group as a numerical value.

CI, 95% confidence interval; OR, odds ratio; PTFQI, Parametric Thyroid Feedback Quantile-based Index; Ref., reference.

The study protocol was approved by the ethical committee of Clinical Research of Aragón (CEICA) (expedient number 19-041).

### PTFQI and thyroid regulation space

For computing the PTFQI, TSH and fT4 are referenced to the standard normal cumulative distribution function (cdf) of the population for these parameters. Then, the difference between the fT4 quantile and the TSH inverse quantile is calculated. The PTFQI formula, as previously described,^11^ is: *φ*((fT4 − *μ*_fT4_)/*σ*_fT4_) − (1 − *φ*((lnTSH − *μ*_lnTSH_)/*σ*_lnTSH_))), where *μ*_fT4_ = 11.49 pmol/L, *σ*_fT4_ = 2.46 pmol/L, *μ*_lnTSH_ = 0.55, and *σ*_lnTSH_ = 1.00 in our sample (see a detailed explanation of PTFQI in the Supplementary Data).

### Thyroid function regulation groups

The bidimensional space defined by the variables TSH and fT4 was used to characterize patients' thyroid regulation.

**FIG. 2.**
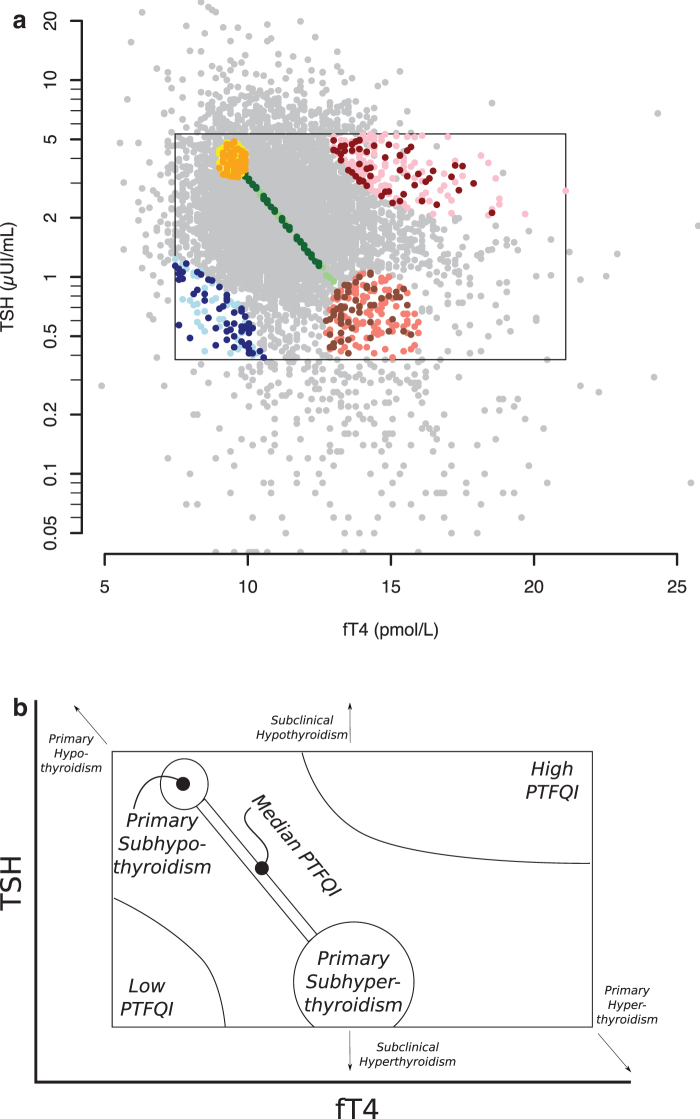
Thyroid function regulation space, comparison groups and selected participants. (**a**) Dots represent participants in the study. The rectangle defines the normal values. Gray dots represent all 6434 analyses. Participants with clinical records reviewed are shown, depending on comparison group assignment, as: blue dots if Low PTFQI, green dots if Median PTFQI, red dots if High PTFQI, yellow dots if Primary Subhypothyroidism, and salmon dots if Primary Subhyperthyroidism. Those selected for detailed analysis after applying exclusion criteria are shown as: dark blue if Low PTFQI, dark green if Median PTFQI, dark red if High PTFQI, orange if Primary Subhypothyroidism, and brown if Primary Subhyperthyroidism. (**b**) Schematic representation of the groups.

Five nonoverlapping groups were defined and sampled for record review (Figs. 1 and 2a, b): *Low PTFQI*, *High PTFQI*, *Median PTFQI* (value close to 0), and *Primary Subhypothyroidism* and *Primary Subhyperthyroidism*. The latter groups are particular cases of PTFQI close to 0 and belonging to them prevented being included in the Median PTFQI group; TSH and fT4 in these latter groups are clinically normal, but should they be more extreme, beyond the limits, they would imply a diagnosis of primary (i.e., glandular) hypothyroidism or hyperthyroidism. Therefore, we used the “sub-” prefix for them. Median PTFQI, Primary Subhypothyroidism, and Primary Subhyperthyroidism conform a *Combined Median PTFQI* group for most analyses, representing the median average response in the pituitary regulation of the thyroid gland (Fig. 1).

The set of screened records in each group was progressively enlarged until at least 50 patients satisfied the selection criteria for detailed analysis, with the aim of having an adequate sample size for the analyses. Details on the creation of the groups are available in the Supplementary Data.

### Laboratory measurements and methods

TSH and fT4 were measured in an automated analyzer by UniCel DxI Beckman's System^®^. Their normal ranges were 0.38–5.33 μUI/mL for TSH and 7.46–21.11 pmol/L for fT4. Total cholesterol, high-density lipoprotein cholesterol (HDL-cholesterol), triglycerides, albumin, and fasting blood glucose were measured with standard enzymatic methods, and low-density lipoprotein cholesterol (LDL-cholesterol) was calculated.

Enzyme activities were measured for aspartate aminotransferase (AST), alanine transaminase (ALT), gamma-glutamyl transpeptidase (GGT), and lactate dehydrogenase (LDH).

The normal reference values from the laboratory were used as cutoff values for dichotomous analyses.

### Clinical data

Clinical records review was structured to collect prespecified data on health and the presence of target diseases. Demographics, diagnoses, physical examinations, and treatments were obtained from the Digital Medical Records System.

### Statistical analysis

Variables are described as mean (SD) or median (interquartile range), or percentage [count] as appropriate. Nonparametric statistical tests were applied when required. The presence of the exclusion criteria (after the first selection step) and the prevalence of type 2 diabetes, atrial fibrillation, ischemic heart disease, and hypertension, and average values of continuous variables (after the second selection step, i.e., the detailed analysis) were described across the PTFQI groups.

Linear and logistic regression models adjusted for age and sex were fitted to estimate odds ratios (ORs) and differences, and trends in prevalences and averages between the groups. A test for trend was used in three-groups-modeling (Low PTFQI, Combined Median PTFQI, and High PTFQI)—significance of a term, introduced as natural number—and a heterogeneity test in the full five-groups-modeling—significance of the linear combination of the dummy variables of the groups. For ischemic heart disease, unadjusted ORs were calculated with the Haldane–Anscombe method to cope with the absence of cases in one of the groups.

We also compared the prevalence of diseases in the groups in the corners of the regulation space (median PTFQI was not used in these analyses), adjusting for age and sex. Pair-wise comparisons (stratified), without testing for significance, provided an exploratory description of the associations. Subsequently, a joint model with variables representing each separated axis (low vs. high TSH and low vs. high fT4) was used to infer the independent association with each parameter. Statistical significance was established at *p* ≤ 0.05. We calculated 95% confidence intervals [CI] for estimates.

All analyses were performed with statistical computing software R version 4.1.

## Results

Among the 661 clinical records reviewed (208 male, 31.5%), exclusion criteria for secondary selection were differently associated with study groups (Table 1), and several exclusion criteria occurred only in a few patients (Supplementary Table S1). Pregnancy was the only physiological condition considered for exclusion, and it was associated with Low PTFQI (*p* < 0.01). Clinical records of 296 patients (108 male, 36.5%) with mean age of 62.8 (16.3) years were selected for the detailed analysis. Age was progressively higher from Low PTFQI to High PTFQI (*p* < 0.01), while sex was not clearly associated with Low or High PTFQI (36.2%, 33.9%, and 45.5% male in the Low, Combined Median, and High PTFQI groups respectively, *p*-Trend = 0.25).

### PTFQI subgroups: Low PTFQI, Combined Median PTFQI, and High PTFQI

After adjusting for age and sex, as the PTFQI increased (three-groups-modeling), there was a statistically significant trend to increase the prevalence of type 2 diabetes (*p* = 0.02), ischemic heart disease (*p* = 0.04), atrial fibrillation (*p* = 0.05), and hypertension (*p* = 0.05) (Table 2). A significant trend was also observed for GGT ≥55 U/L (*p* = 0.02), LDH ≥248 U/L (*p* = 0.02), and albumin ≤3.5 g/dL (*p* = 0.03). Continuous variables analyzed showed a statistically significant PTFQI-dependent trend in pulse pressure (direct, *p* = 0.01), creatinine (direct, *p* = 0.01), total cholesterol (inverse, *p* < 0.01), AST (direct, *p* < 0.01), GGT (direct, *p* = 0.02), and LDH (direct, *p* = 0.04) (Table 3).

**Table 2.** Presence of Categorical Variables According to Thyroid Regulation Groups

Variables	*N*	Low PTFQI	Combined median PTFQI			High PTFQI	*p*-Heter 5 groups	*p*-Trend 3 groups
			Primary subhypothyroidism	Median PTFQI	Primary subhyperthyroidism			
		% [*n*]	% [*n*]	% [*n*]	% [*n*]	% [*n*]		
		(*N* = 58)	(*N* = 71)	(*N* = 53)	(*N* = 59)	(*N* = 55)		
Type 2 diabetes	296	13.8 [8]	26.8 [19]	24.5 [13]	27.1 [16]	41.8 [23]		
		1.00 (Ref.)	1.89 [0.76–5.09]	1.58 [0.58–4.46]	1.51 [0.58–4.19]	2.88 [1.14–7.86]	0.23	0.02
Ischemic heart disease	296	0.0 [0]	8.5 [6]	11.3 [6]	15.3 [9]	16.4 [9]		
		1.00 (Ref.)	—	—	—	—	0.03	0.04
Atrial fibrillation	296	1.7 [1]	9.9 [7]	7.5 [4]	20.3 [12]	21.8 [12]		
		1.00 (Ref.)	4.64 [0.72–91.99]	3.73 [0.48–77.92]	8.12 [1.33–157.86]	8.13 [1.33–158.20]	0.13	0.05
Cerebrovascular disease	296	3.4 [2]	2.8 [2]	11.3 [6]	3.4 [2]	14.5 [8]		
		1.00 (Ref.)	0.52 [0.06–4.67]	2.38 [0.48–17.42]	0.44 [0.05–3.98]	2.21 [0.47–15.88]	0.08	0.15
COPD	296	0.0 [0]	0.0 [0]	3.8 [2]	6.8 [4]	0.0 [0]		
		1.00 (Ref.)	—	—	—	—	0.02	0.81
OSAS	296	0.0 [0]	5.6 [4]	7.5 [4]	8.5 [5]	5.5 [3]		
		1.00 (Ref.)	—	—	—	—	0.10	0.23
Hypertension (Exam BP ≥140/90)	254	14.3 [6]	28.6 [18]	36.7 [18]	38.9 [21]	41.3 [19]		
		1.00 (Ref.)	2.27 [0.84–6.92]	3.16 [1.14–9.78]	2.99 [1.10–9.17]	3.19 [1.14–9.94]	0.16	0.05
Obesity (Exam BMI ≥30 kg/m^2^)	296	22.4 [13]	36.6 [26]	37.7 [20]	28.8 [17]	29.1 [16]		
		1.00 (Ref.)	1.82 [0.83–4.12]	2.00 [0.87–4.73]	1.32 [0.56–3.17]	1.34 [0.55–3.28]	0.45	0.51
Overweight (Exam BMI ≥25 kg/m^2^)	296	44.8 [26]	59.2 [42]	67.9 [36]	57.6 [34]	56.4 [31]		
		1.00 (Ref.)	1.60 [0.79–3.29]	2.32 [1.07–5.18]	1.37 [0.64–2.95]	1.27 [0.59–2.78]	0.29	0.50
Dyslipidemia^a^	296	48.3 [28]	57.7 [41]	50.9 [27]	49.2 [29]	63.6 [35]		
		1.00 (Ref.)	1.10 [0.51–2.37]	0.75 [0.33–1.69]	0.53 [0.23–1.19]	0.97 [0.42–2.23]	0.36	0.99
Hypertriglyceridemia (≥200 mg/dL)	296	5.2 [3]	14.1 [10]	20.8 [11]	3.4 [2]	9.1 [5]		
		1.00 (Ref.)	2.92 [0.82–13.81]	4.34 [1.23–20.41]	0.52 [0.06–3.31]	1.43 [0.32–7.50]	0.01	0.73
Hypoalbuminemia (≤3.5g/dL)	296	3.4 [2]	4.2 [3]	1.9 [1]	11.9 [7]	18.2 [10]		
		1.00 (Ref.)	0.98 [0.15–7.73]	0.43 [0.02–4.65]	2.55 [0.55–18.17]	4.11 [0.95–28.53]	0.04	0.03
AST ≥50 U/L	293	3.5 [2]	2.9 [2]	1.9 [1]	1.7 [1]	7.3 [4]		
		1.00 (Ref.)	0.83 [0.10–7.23]	0.58 [0.03–6.39]	0.55 [0.02–6.30]	2.62 [0.44–21.39]	0.50	0.22
ALT ≥50 U/L	296	8.6 [5]	5.6 [4]	1.9 [1]	0.0 [0]	5.5 [3]		
		1.00 (Ref.)	—	—	—	—	0.07	0.44
GGT ≥55 U/L	260	13.0 [6]	14.3 [9]	9.8 [5]	6.1 [3]	29.4 [15]		
		1.00 (Ref.)	1.16 [0.38–3.73]	0.73 [0.19–2.61]	0.43 [0.08–1.78]	2.73 [0.96–8.62]	0.02	0.02
LDH ≥248 U/L	243	7.5 [3]	6.8 [4]	10.6 [5]	8.5 [4]	22.0 [11]		
		1.00 (Ref.)	0.85 [0.18–4.58]	1.50 [0.34–7.82]	1.22 [0.24–6.76]	3.81 [1.02–18.68]	0.11	0.02

Data are expressed in % [*n*]. OR [CI] from models adjusted for age and sex, referenced to the Low PTFQI group. *p*-Heter is the *p*-value for testing differences among groups. *p*-Trend is the *p*-value from a regression model that includes group as a numerical value.

^a^ Included being treated with lipid-lowering drugs.

ALT, alanine transaminase; AST, aspartate transaminase; BMI, body mass index; BP, blood pressure; COPD, chronic obstructive pulmonary disease; GGT, gamma-glutamyl transpeptidase; LDH, lactate dehydrogenase; OSAS, obstructive sleep apnea syndrome.

**Table 3.** Means or Medians of Continuous Quantitative Variables According to Thyroid Regulation Groups

Variables	*N*	Low PTFQI	Combined median PTFQI			High PTFQI	*p*-Heter 5 groups	*p*-Trend 3 groups
			Primary subhypothyroidism	Median PTFQI	Primary subhyperthyroidism			
		% [*n*]	% [*n*]	% [*n*]	% [*n*]	% [*n*]		
		(*N* = 58)	(*N* = 71)	(*N* = 53)	(*N* = 59)	(*N* = 55)		
Age	296	54.3 (16.3)	61.2 (15.9)	61.8 (14.7)	66.3 (16.1)	67.6 (15.4)		
		0.00 (Ref.)	6.64 [1.19 to 12.10]	7.48 [1.63 to 13.32]	12.03 [6.34 to 17.72]	13.47 [7.67 to 19.26]	<0.01	<0.01
Weight	247	75.6 (16.5)	75.9 (17.2)	76.7 (15.3)	75.1 (19.3)	73.7 (14.8)		
		0.00 (Ref.)	0.79 [−5.47 to 7.06]	0.78 [−5.86 to 7.41]	−0.55 [−7.18 to 6.08]	−1.93 [−8.71 to 4.85]	0.92	0.46
Height	240	164.9 (7.4)	161.5 (9.1)	162.9 (8.2)	162.4 (11.2)	162.5 (9.6)		
		0.00 (Ref.)	−2.97 [−5.56 to −0.38]	−2.24 [−4.96 to 0.49]	−2.05 [−4.77 to 0.68]	−2.22 [−4.99 to 0.54]	0.26	0.09
BMI	240	27.7 (5.5)	29.3 (6.1)	28.9 (5.8)	28.7 (7.6)	27.9 (5.4)		
		0.00 (Ref.)	1.56 [−0.92 to 4.04]	1.08 [−1.53 to 3.68]	0.91 [−1.69 to 3.52]	0.12 [−2.53 to 2.77]	0.70	0.97
SBP	254	125.5 (14.3)	127.8 (18.8)	132.5 (16.2)	132.9 (16.6)	134.8 (18.2)		
		0.00 (Ref.)	1.69 [−4.76 to 8.14]	5.58 [−1.25 to12.41]	4.56 [−2.21 to 11.33]	6.03 [−1.02 to 13.09]	0.34	0.08
DBP	254	77.4 (8.5)	74.9 (9.6)	77.0 (10.3)	76.0 (10.4)	74.5 (9.6)		
		0.00 (Ref.)	−2.25 [−6.03 to 1.53]	−0.12 [−4.12 to 3.88]	−0.77 [−4.74 to 3.19]	−2.13 [−6.27 to 2.00]	0.64	0.26
Pulse pressure	254	48.1 (11.2)	53.0 (15.7)	55.5 (14.4)	56.9 (16.2)	60.3 (17.1)		
		0.00 (Ref.)	3.94 [−1.56 to 9.44]	5.71 [−0.12 to 11.53]	5.33 [−0.44 to 11.11]	8.17 [2.15 to 14.18]	0.11	0.01
Urea^*^	294	34.0 (29.0, 42.8)	34.0 (29.0, 42.0)	35.0 (29.0, 43.0)	38.0 (32.2, 45.8)	39.0 (32.2, 48.8)	<0.01^a^	0.06^b^
Creatinine^*^	296	0.7 (0.6, 0.9)	0.8 (0.6, 0.9)	0.8 (0.6, 1.0)	0.8 (0.6, 0.9)	0.9 (0.7, 1.1)	<0.01^a^	0.01^b^
Uric acid	294	5.2 (1.5)	5.6 (1.6)	5.6 (1.6)	5.5 (2.0)	5.9 (1.7)		
		0.00 (Ref.)	0.37 [−0.19 to 0.94]	0.27 [−0.33 to 0.88]	−0.02 [−0.62 to 0.57]	0.31 [−0.30 to 0.93]	0.52	0.19
Total cholesterol	296	202.5 (37.7)	196.1 (36.0)	197.9 (35.9)	186.3 (41.7)	180.3 (48.9)		
		0.00 (Ref.)	−8.40 [−22.14 to 5.33]	−4.42 [−19.14 to 10.31]	−14.58 [−29.18 to 0.02]	−19.90 [−34.86 to −4.94]	0.07	<0.01
HDL-cholesterol	241	54.4 (13.5)	49.8 (13.4)	50.0 (12.9)	50.5 (11.4)	50.3 (12.4)		
		0.00 (Ref.)	−3.77 [−8.60 to 1.05]	−3.82 [−8.97 to 1.32]	−2.10 [−7.22 to 3.02]	−1.66 [−6.94 to 3.62]	0.521	0.680
Triglycerides^*^	296	96.0 (75.0, 132.8)	113.0 (81.0, 155.5)	109.0 (74.0, 180.0)	95.0 (73.0, 127.0)	121.0 (79.0, 138.5)	0.85^a^	0.19^b^
LDL-cholesterol	232	127.6 (31.7)	119.4 (31.5)	117.2 (27.9)	113.6 (38.9)	110.7 (44.7)		
		0.00 (Ref.)	−5.82 [−19.38 to 7.75]	−8.85 [−23.25 to 5.55]	−8.11 [−22.26 to 6.04]	−9.46 [−24.30 to 5.38]	0.70	0.08
NoHDL-cholesterol	240	148.9 (33.9)	145.9 (35.4)	144.5 (32.3)	135.3 (40.6)	134.2 (45.9)		
		0.00 (Ref.)	−1.54 [−15.98 to 12.90]	−3.30 [−18.68 to 12.08]	−9.41 [−24.82 to 5.99]	−9.32 [−25.11 to 6.47]	0.66	0.11
AST^*^	293	21.0 (19.0, 28.0)	22.0 (18.0, 27.8)	21.5 (18.0, 25.2)	19.0 (16.0, 23.5)	25.0 (19.5, 30.5)	0.89^a^	<0.01^b^
ALT^*^	296	19.0 (14.2, 28.0)	19.0 (13.0, 26.0)	17.0 (14.0, 22.0)	17.0 (12.5, 23.0)	21.0 (14.0, 29.5)	0.86^a^	0.20^b^
GGT^*^	260	22.5 (17.2, 33.8)	21.0 (17.0, 33.0)	22.0 (16.0, 34.5)	23.0 (16.0, 31.0)	30.0 (20.0, 76.5)	0.07^a^	0.03^b^
LDH^*^	243	186.5 (158.5, 214.5)	186.0 (169.0, 207.5)	188.0 (162.0, 210.5)	167.0 (146.5, 217.0)	200.0 (171.5, 244.8)	0.24^a^	0.05^b^
Fasting glucose^*^	296	94.5 (88.2, 105.2)	92.0 (87.0, 104.0)	96.0 (88.0, 111.0)	100.0 (87.0, 114.0)	97.0 (84.0, 132.5)	0.21^a^	0.71^b^
HbA1c^*^	106	5.8 (5.5, 6.4)	6.2 (5.5, 7.3)	6.0 (5.8, 7.1)	6.4 (5.8, 7.3)	6.1 (5.5, 7.2)	0.53^a^	0.70^b^
Hematocrit	262	42.1 (3.1)	41.4 (3.9)	41.9 (3.5)	41.8 (4.2)	41.1 (5.1)		
		0.00 (Ref.)	−0.09 [−1.47 to 1.29]	0.18 [−1.31 to 1.67]	−0.17 [−1.61 to 1.27]	−0.90 [−2.37 to 0.58]	0.66	0.27
Hemoglobin	262	14.1 (1.1)	13.8 (1.4)	14.0 (1.3)	14.0 (1.5)	13.7 (1.9)		
		0.00 (Ref.)	−0.04 [−0.52 to 0.45]	0.05 [−0.47 to 0.58]	−0.02 [−0.52 to 0.49]	−0.32 [−0.84 to 0.20]	0.64	0.27
Red blood cells	262	4.7 (0.4)	4.6 (0.5)	4.7 (0.4)	4.7 (0.5)	4.6 (0.6)		
		0.00 (Ref.)	0.01 [−0.16 to 0.17]	0.07 [−0.11 to 0.25]	−0.01 [−0.18 to 0.17]	−0.08 [−0.26 to 0.09]	0.59	0.39
White blood cells	262	6.9 (2.0)	7.1 (1.8)	7.4 (2.5)	7.2 (2.1)	7.2 (2.2)		
		0.00 (Ref.)	0.33 [−0.45 to 1.11]	0.61 [−0.23 to 1.46]	0.43 [−0.38 to 1.24]	0.42 [−0.41 to 1.25]	0.69	0.38
Lymphocytes	262	31.6 (9.3)	34.8 (9.4)	32.1 (7.4)	28.8 (10.2)	30.8 (8.8)		
		0.00 (Ref.)	2.90 [−0.52 to 6.32]	0.35 [−3.35 to 4.06]	−2.87 [−6.44 to 0.70]	−0.79 [−4.44 to 2.86]	0.02	0.75
Neutrophils	262	56.8 (10.3)	52.8 (9.4)	55.6 (9.0)	59.9 (11.5)	56.8 (8.9)		
		0.00 (Ref.)	−3.64 [−7.34 to 0.06]	−0.94 [−4.95 to 3.07]	3.57 [−0.30 to 7.43]	0.48 [−3.47 to 4.43]	<0.01	0.88

Data are expressed in mean (standard deviation). For nonnormal variables (^*^), data are expressed in median and interquartile range. Adjusted means [CI] from models adjusted for age and sex, referenced to the Low PTFQI group. *p*-Heter is the *p*-value for testing differences among groups. *p*-Trend is the *p*-value from a regression model that includes group as a numerical value.

^*^ Nonparametric variables.

^a^ Kruskal–Wallis test.

^b^ Spearman's Rho test.

DBP, diastolic blood pressure; HDL, high-density lipoprotein; HbA1c, glycosylated hemoglobin; LDL, low-density lipoprotein; Ref., reference; SBP, systolic blood pressure.

Missing values of continuous variables were not associated with the PTFQI groups with the exception of LDH, which was more frequently measured in lower PTFQI groups (*p* = 0.02). Treatments within pre-established groups (beta-blockers, *p* = 0.35, antihypertensive drugs, *p* = 0.40, lipid-lowering drugs, *p* = 0.50, and antidepressant/anxiolytic drugs, *p* = 0.48) were not associated with the PTFQI groups (Supplementary Table S2).

### Primary subhypothyroidism and subhyperthyroidism subgroups

From the Combined Median PTFQI group, we segregated those patients who had feedback-consistent congruent variation of TSH and fT4. We tested for heterogeneity across the resultant groups (five-groups-modeling). Statistical significance was reached for ischemic heart disease (*p* = 0.03) and hypertriglyceridemia (*p* = 0.01) (Table 2) and for urea (*p* < 0.01), creatinine (*p* < 0.01), and the percentage of lymphocytes (*p* = 0.02) and neutrophils (*p* < 0.01) (Table 3).

### Different dependence on TSH or fT4

**FIG. 3.**
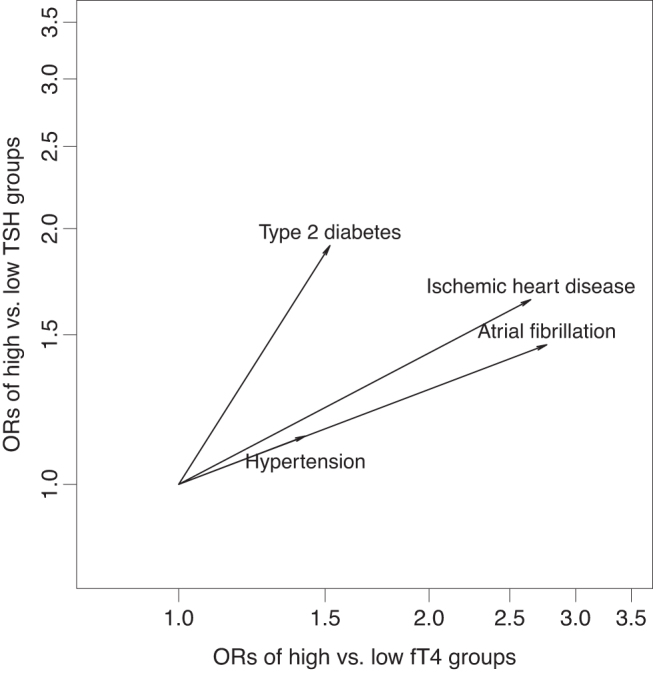
Association of diseases across both thyroid axes: influence of TSH and fT4 after mutual adjustment. The arrow lengths across each axis show ORs for each disease of high vs. low group on each axis (fT4 and TSH), from an age- and sex-adjusted model that includes both. Note that type 2 diabetes depends more on TSH, while ischemic heart disease, atrial fibrillation, and hypertension depend more on fT4. fT4, free thyroxine; ORs, odds ratios; TSH, thyrotropin.

As an exploratory analysis, we compared type 2 diabetes, ischemic heart disease, atrial fibrillation, and hypertension prevalence pair-wise in the groups in the corners of the regulation space (Supplementary Table S3). In the joint model, increases in both variables were associated with increases in disease prevalences but we observed that type 2 diabetes was more influenced by TSH (OR_TSH_: 1.91 [CI: 1.04–3.56] and OR_fT4_: 1.52 [CI: 0.82–2.84]) while ischemic heart disease (OR_TSH_: 1.65 [CI: 0.68–4.14] and OR_fT4_: 2.65 [CI: 1.05–7.24]), atrial fibrillation (OR_TSH_: 1.46 [CI: 0.61–3.58] and OR_fT4_: 2.77 [CI: 1.11–7.36]), and hypertension (OR_TSH_: 1.14 [CI: 0.62–2.08] and OR_fT4_: 1.42 [CI: 0.76–2.64]) were more influenced by fT4 (Fig. 3).

Given the reduced size of the subgroups, next, we enumerate the following findings as descriptive results. There is an interaction, not statistically significant, between TSH and fT4 for ischemic heart disease and atrial fibrillation (Supplementary Table S3) where the most association appears when TSH or fT4 rise in isolation while they do not sum up their effects. On the contrary, hypertension seems to be barely affected unless both parameters rise. In these stratified analyses, statistical significance could only be proved for atrial fibrillation, in the transition from the Low PTFQI group to the Subhyperthyroidism group (in the low TSH stratum across the fT4 axis) (Supplementary Table S3).

## Discussion

In this sample of ambulatory patients, we found an association between upregulated thyroid axis (high PTFQI) and type 2 diabetes, ischemic heart disease, atrial fibrillation, hypertension, and other clinical parameters. The PTFQI quantifies the thyroid regulation set point.^11^ This is the first description of the association of high PTFQI with ischemic heart disease and atrial fibrillation.

### High PTFQI and diabetes

Subclinical hypothyroidism affects insulin secretion and impairs vascular function, increasing diabetic nephropathy and retinopathy risk, as well as peripheral arterial disease and neuropathy;^12^ excess circulating thyroid hormone in hyperthyroidism also associates with poor glycemic control and endothelial dysfunction, increasing cardiovascular complications.^12^ Among euthyroid subjects, fT4 close to the upper limit of normality associates to more frequent type 2 diabetes^2^ while TSH and type 2 diabetes do not show a clear linear relationship. Our results agree with previous reports associating hyperthyroxinemia or lower sensitivity to thyroid hormone and type 2 diabetes in two other independent populations.^11,13^

Perhaps sensitivity to thyroid hormone is impaired in the central and regulatory structures, as well as in peripheral tissues. While in the former, it implies a high PTFQI, in the latter, it would favor obesity, diabetes, and metabolic syndrome. Another explanation could be that an excess in energy intake induces as a compensatory mechanism an elevation in the pituitary set point. We show that the magnitude of association of diabetes with TSH is similar to that with fT4, emphasizing that the upregulation as captured by PTFQI is related to a significantly higher diabetes prevalence.

### High PTFQI and cardiovascular disease

The association of thyroid dysfunction with cardiovascular disease has been thoroughly addressed.^14–17^ Either excessive or deficient function can induce or exacerbate cardiovascular disorders, such as coronary disease, hypertension, and arrhythmias, especially of atrium origin.

The most common cardiovascular complication of hyperthyroidism is atrial fibrillation,^18^ which can occur with higher fT4, even within the normal range.^9,19^ However, its association with TSH within its normal range is not clearly linear.^9^ Our study suggests that a concomitant elevation of TSH and fT4, that is, higher PTFQI, is associated with atrial fibrillation. An acquired impaired sensitivity in the receptor β pathway^3^ could be a possible explanation. In fact, genetic resistance to thyroid hormone affecting receptor β produces central resistance and metabolic effects such as diabetes, but conserved, even increased thyroid effects dependent on receptor α, such as heart chronotropism.^20^ Alternatively, if higher PTFQI is interpreted as a central upregulation of the thyroid axis, a TSH rise is followed by a persistent increase in fT4, exerting its chronotropic effect on the heart.

Patients who have clinical or subclinical hyperthyroidism have a higher degree of coronary stenosis and cardiovascular mortality.^14,17^ Elevation of fT4 is hypothesized to influence vascular degeneration and instability of atheroma plaques.^14^ Bringing complication to this matter, ischemic heart disease is also affected by hypothyroidism, where dyslipidemia increases the risk of acute myocardial infarction.^21–23^ Interestingly, it has been proposed that psychosocial stress may contribute to cardiovascular risk via an increased set point of thyroid homeostasis.^24^ We found that ischemic heart disease rose with PTFQI in euthyroid subjects. It might depend mostly on fT4 concentration, as we found, but association of coronary heart disease with fT4 (alone) across the euthyroid range could not be demonstrated so far.^7,8^ It is remarkable the absence of ischemic heart disease among patients in the Low PTFQI group, a fact that may well deserve future in-depth research.

Thyroid hormone contributes to pressure regulation, and hypertension appears in hyperthyroidism as well as hypothyroidism. Furthermore, hypertension may be the initial clinical presentation for a thyroid disorder.^25^ Hyperthyroidism increases systolic blood pressure, decreases diastolic blood pressure, and widens pulse pressure, while hypothyroidism acts oppositely.^22^ We found that hypertension and high pulse pressure were increased in high PTFQI. As Abdi et al^26^ already explained, we have found that in euthyroid patients, blood pressure is more regulated by fT4 than by TSH. Other cross-sectional and cohort studies^27,28^ also support a higher association with fT4. However, our stratified analysis suggested a tendency for synergy of TSH and fT4 on hypertension.

Interestingly, in this work, we demonstrate that thyroid upregulation appears in both diabetes and cardiovascular disease. This could be one more pathophysiological mechanism to account for among the links between diabetes and cardiovascular disease.

### Potential clinical importance of this research

The PTFQI, as a marker of an abnormal metabolic state, could serve to identify subjects at risk allowing preventive strategies. Potentially, it could also be used to monitor treatments. With respect to atrial fibrillation, there is controversy regarding the normal limits for fT4 since mostly patients with atrial fibrillation have fT4 in its upper-normal limits. However, the PTFQI and its relationship with atrial fibrillation was not previously explored and could provide a more detailed approach in identifying patients who would benefit from modifying their therapies.

### Limitations

This study is performed with ambulatory patients who, for various reasons, underwent thyroid hormone measurements. Therefore, this must be taken into account when interpreting results, as the sample is not representative of the general population. However, we believe that it is suitable for the cross-sectional analysis that we performed. The regulation space was sampled only at the studied groups. Nevertheless, there is no reason to believe that intermediate regulation values (not sampled) do not have also intermediate distributions of the diseases that we found, as it is suggested by the described gradient. To which extent this regulation variable, PTFQI, associates with the prevalence of each disease in the general population, as attributable risk, remains a matter for future research, including data from other populations.

As we performed multiple exploratory statistical tests, some of the statistically significant associations may be false positives. Conversely, the sample size, limited by the feasibility of the review of clinical records, implies that statistical significance was not reached in some analyses despite an apparent tendency in the association. Anthropometric data and blood pressure values available in patients' medical records often were not simultaneous to the determination of the hormones. However, although it has not been possible to adjust for body mass index in this sample, we have previously shown that the association of high PTFQI with diabetes is independent of body mass index.^11^

Another important limitation is that free triiodothyronine (fT3) levels were not available, so the inhibition of TSH synthesis by fT3 has not been considered. Pituitary deiodinases, as part of thyroid synthesis and regulation, have an important role. Their influence was considered to be conveyed within our measurement as regulation occurring downstream of fT4. While less active deiodinases could justify high fT4 levels due to a low conversion to its most active form, fT3, greater deiodinase activity could be related to greater thyroid sensitivity, with lower fT4 serum levels. Finally, the cross-sectional nature of our study does not allow establishing causal relationships. Those will need to be confirmed in prospective studies.

## Conclusions

In conclusion, an elevation of the pituitary set point of the thyroid axis, measured by PTFQI, was found to be associated with increased prevalence of type 2 diabetes, atrial fibrillation, ischemic heart disease, and hypertension in euthyroid adults. These findings, which highlight the importance of thyroid regulation implications in non-thyroid diseases in euthyroid subjects, should be confirmed in other populations.

## Supplementary Material

Supplemental data

## Data Availability

The data sets used and/or analyzed during the current study are available from the corresponding author on reasonable request.
