# Supplementary material for: A Cross-Sectional Study Examining the Parametric Thyroid Feedback Quantile Index and Its Relationship with Metabolic and Cardiovascular Diseases
Source: Thyroid. 2022 Dec 13;32(12):1488–99. doi: 10.1089/thy.2022.0025 (PMC9807248; doi:10.1089/thy.2022.0025)
Supplement: Supplemental data [file Supp_Data.pdf]

Alonso-Ventura V, Civeira F, Alvarado-Rosas A, Lou-Bonafonte JM, Calmarza P, Moreno-Franco B, Andres-Otero MJ, Calvo-Gracia F, de Diego-Garcia P, Laclaustra M.

A cross-sectional study examining the Parametric Thyroid Feedback Quantile Index and its relationship with metabolic and cardiovascular diseases

THYROID

2022

DOI: 10.1089/thy.2022.0025

SUPPLEMENTARY MATERIAL

Alonso-Ventura V, Civeira F, Alvarado-Rosas A, Lou-Bonafonte JM, Calmarza P, Moreno-Franco B, Andres-Otero MJ, Calvo-Gracia F, de Diego-Garcia P, Laclaustra M. A cross-sectional study examining the Parametric Thyroid Feedback Quantile Index and its relationship with metabolic and cardiovascular diseases. Thyroid. 2022. doi: 10.1089/thy.2022.0025

METHODS: Exclusion criteria

- Being pregnant
- Being on treatment with levothyroxine
- Being on treatment with antithyroid drugs
- Having pre-existent thyroid disorders or thyroid physical therapies evidenced by recorded diagnosis or by previously deranged thyroid analytical values
    - Pre-existent thyroid disorders
    - Positive antithyroid antibodies
    - Thyroidectomy for cancer
    - Thyroidectomy for goiter
    - Treatment with radioactive iodine
- Being on treatment with amiodarone
- Being on treatment with steroids; or, at least, having been on treatment in the year before the laboratory analysis
- Having an endocrine syndrome which involves directly or indirectly the thyroid axis
    - Cushing Syndrome
    - Addison's Disease
    - Turner and Klinefelter Syndromes
    - Type 1 Diabetes
- Situations of organic stress like terminal diseases, advanced disease stages, or aggressive treatments affecting the thyroid axis
    - Hospitalization
    - Hemodialysis
    - Radiotherapy in neck

Alonso-Ventura V, Civeira F, Alvarado-Rosas A, Lou-Bonafonte JM, Calmarza P, Moreno-Franco B, Andres-Otero MJ, Calvo-Gracia F, de Diego-Garcia P, Laclaustra M. A cross-sectional study examining the Parametric Thyroid Feedback Quantile Index and its relationship with metabolic and cardiovascular diseases. Thyroid. 2022. doi: 10.1089/thy.2022.0025

METHODS: CREATION OF GROUPS SAMPLED IN THE THYROID REGULATION SPACE

Group creation was performed sequentially: The three PTFQI groups (Low PTFQI, High PTFQI, and Median PTFQI) were created first and their targeted records were reviewed, starting from those with PTFQI most extreme values and adding records towards the median. To select the remaining two groups (Primary Subhypothyroidism and Primary Subhyperthyroidism), TSH and fT4 averages from participants in Low PTFQI and High PTFQI groups who fulfilled selection criteria for the detailed analysis, once their records were reviewed, were projected to the median PTFQI line (PTFQI = 0) in order to define center TSH and fT4 values for the Primary Subhypothyroidism and Primary Subhyperthiroidism groups, which purpose is to provide positive comparability in particular statistical analyses.

Alonso-Ventura V, Civeira F, Alvarado-Rosas A, Lou-Bonafonte JM, Calmarza P, Moreno-Franco B, Andres-Otero MJ, Calvo-Gracia F, de Diego-Garcia P, Laclaustra M. A cross-sectional study examining the Parametric Thyroid Feedback Quantile Index and its relationship with metabolic and cardiovascular diseases. Thyroid. 2022. doi: 10.1089/thy.2022.0025

METHODS: PTFQI RATIONALE AND CALCULATION METHOD

1. **Rationale of the index**

In an idealized population where regulation is identical and uniform across all individuals, the highest fT4 would be accompanied by the lowest TSH. The next top fT4 by the second lowest TSH, and so forth. In statistics the position number of individuals after ordering by continuous variables is called rank and its mapping to a 0-1 interval is called quantile. In the mentioned population, fT4 quantiles would be the exact reversal of TSH quantiles. For a particular individual in a real population the differences between fT4 quantile and the reversed TSH quantile informs on how much this particular subject differs from the ideal regulation as expected in the population.

When all the subjects of the population are available, real quantiles within the sample can be calculated which are used by the Thyroid Feedback Quantile-based Index (TFQI). If what is available is the descriptors of central tendency and dispersion of fT4 and TSH (logarithmically transformed), the quantile can be estimated for a single individual, assuming a normal distribution of these two variables.

2. **Calculation**

PTFQI formula: $\phi((fT4-\mu_{fT4})/\sigma_{fT4})-(1-\phi((lnTSH-\mu_{lnTSH})/\sigma_{lnTSH})))$

ϕ (phi) means cumulative standarized normal function

PTFQI can be calculated with the spreadsheet formula

```
=NORM.DIST(fT4_cell_in_pmol_per_L, cell_with_fT4_population_mean,
cell_with_fT4_population_sd, TRUE) + NORM.DIST(LN(TSH_cell_in_mIU_per_L),
cell_with_lnTSH_population_mean, cell_with_lnTSH_population_sd, TRUE) – 1
```

See the accompanying plot.

3. **Interpretation**

PTFQI ranges from -1 to 1: negative values indicate an abnormally low TSH for fT4 values (a down-regulated thyroid set-point) and positive values indicate an abnormally high TSH given fT4 levels (an up-regulated thyroid set-point). Extreme deviations would be compatible with secondary hypo- and hyperthyroidism. Values around 0 mean a nearly perfect regulation, where TSH and fT4 values are coherently regulated.

Alonso-Ventura V, Civeira F, Alvarado-Rosas A, Lou-Bonafonte JM, Calmarza P, Moreno-Franco B, Andres-Otero MJ, Calvo-Gracia F, de Diego-Garcia P, Laclaustra M. A cross-sectional study examining the Parametric Thyroid Feedback Quantile Index and its relationship with metabolic and cardiovascular diseases. Thyroid. 2022. doi: 10.1089/thy.2022.0025

4. **Advantages with respect to other indices**

As we discovered in our previous investigation (Diabetes Care 2019), previous indices of thyroid sensitivity were only suitable to detect extreme thyroid resistance, in patients with genetic defects of receptors. They were devised for other purposes, and the association with diabetes, investigated in that project, was more modest. PTFQI, per definition, measures how the central set-point deviates from the average population regulation. Thus, its intended use is in the general population, while other indices were developed mainly to identify subjects with defects in the thyroid disease signal pathway. Furthermore, by using the origin population parameters as reference, this index can adapt to the different thyroid laboratory methods used and ethnic particularities of thyroid regulation, which makes it more suitable for population studies. PTFQI values are theoretically comparable across populations.

5. **Intrinsic validity**

PTFQI is a new index that we have created to investigate thyroid regulation in the general population. It serves to describe where the central set-point is. Per definition, the index measures discordance between TSH inhibition and fT4 values. So, it is defined by what it measures: it measures the set-point and there is not space for validation against other measurements (as it measures directly the gold-standard: “where is TSH with respect to fT4?”). External acceptance will depend on the clinical utility which will not happen until research results are disseminated.

6. **Recommended application**

We recommend using PTFQI whenever possible, always accompanied with the details reporting the population reference numerical values used: Mean values of fT4 and neper-log of TSH and their standard deviation for the sample (representative of the source population). These would be the values used to calculate PTFQI for every participant with the above-mentioned formula. This improves the applicability of the research. Clinical future local patients from the same population and who share the same lab methods can calculate their indices and refer directly to the researchers’ results. It also improves comparability with further research on the topic.

Alonso-Ventura V, Civeira F, Alvarado-Rosas A, Lou-Bonafonte JM, Calmarza P, Moreno-Franco B, Andres-Otero MJ, Calvo-Gracia F, de Diego-Garcia P, Laclaustra M. A cross-sectional study examining the Parametric Thyroid Feedback Quantile Index and its relationship with metabolic and cardiovascular diseases. Thyroid. 2022. doi: 10.1089/thy.2022.0025

On the contrary, TFQI can only be calculated within one study, having access to the individual data of every participant. We recommend restricting the use of TFQI for closed populations in which assumptions of normality could not be made.

7. Example

In our reference source population:

μ_{fT4} = 11.49 pmol/L and σ_{fT4} = 2.46 pmol/L

μ_{lnTSH} = 0.55 and σ_{lnTSH} = 1.00

A participant with fT4 = 14.10 pmol/L and TSH = 4.3 µUI/mL
We calculate lnTSH = ln(4.3) = 1.459
Z score for fT4 = (14.10 – 11.49) / 2.46 = 1.061
Z score for lnTSH = (1.459 – 0.55) / 1.00 = 0.909
Percentile on fT4 distribution = P85.6
Quantile fT4 = 0.856
Percentile on lnTSH distribution = P81.8
Quantile lnTSH = 0.818
(1 – Quantile ln TSH) = 0.182
PTFQI = Quantile fT4 – (1 – Quantile ln TSH) = 0.856 – 0.182 = 0.674

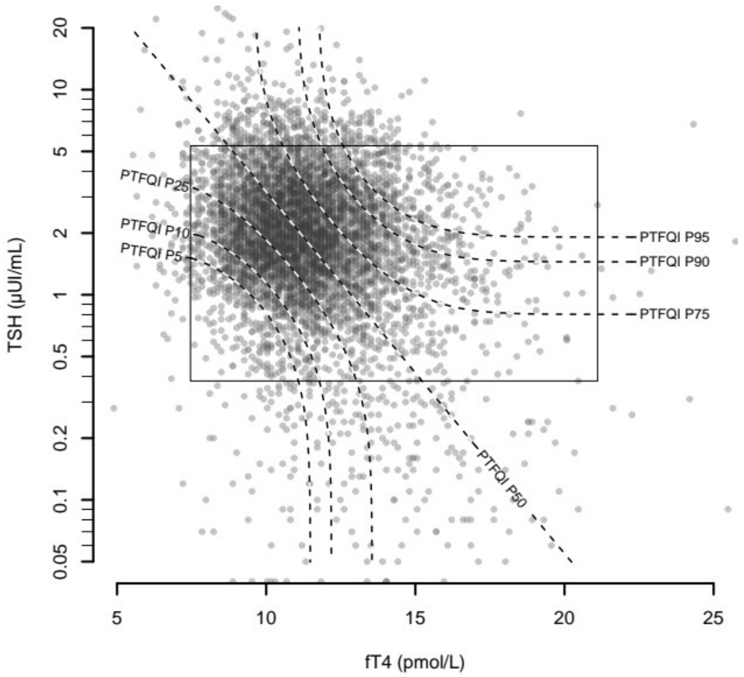

Alonso-Ventura V, Civeira F, Alvarado-Rosas A, Lou-Bonafonte JM, Calmarza P, Moreno-Franco B, Andres-Otero MJ, Calvo-Gracia F, de Diego-Garcia P, Laclaustra M. A cross-sectional study examining the Parametric Thyroid Feedback Quantile Index and its relationship with metabolic and cardiovascular diseases. Thyroid. 2022. doi: 10.1089/thy.2022.0025

Supplementary Table 1. Exclusion criteria by thyroid regulation groups (minor).

EXCLUSION CRITERIA	N excluded	LOW PTFQI	COMBINED MEDIAN PTFQI			HIGH PTFQI
			PRIMARY SUBHYPOTHYROIDISM	MEDIANPTFQI	PRIMARY SUBHYPERTHYROIDISM	
	n/N	%[n] (N=100)	%[n] (N=120)	%[n] (N=77)	%[n] (N=170)	%[n] (N=194)
	25/661	5.0[5]	4.2[5]	1.3[1]	4.7[8]	3.1[6]
Treatment with antithyroid drugs	9/661	3.0[3]	0.8[1]		2.4[4]	0.5[1]
Advanced diseases	7/661		1.7[2]	1.3[1]		2.1[4]
Addison’s disease	2/661				1.2[2]	
Cushing syndrome	2/661	1.0[1]			0.6[1]	
Antithyroid antibodies	1/661		0.8[1]			
Hemodialysis	1/661	1.0[1]				
Klinefelter syndrome	1/661		0.8[1]			
Radiotherapy in neck	1/661				0.6[1]	
Turner syndrome	1/661					0.5[1]

Data expressed in% [n]. Minor exclusion criteria distributed by thyroid regulation groups. PTFQI: Parametric Thyroid Feedback Quantile-based index.

Alonso-Ventura V, Civeira F, Alvarado-Rosas A, Lou-Bonafonte JM, Calmarza P, Moreno-Franco B, Andres-Otero MJ, Calvo-Gracia F, de Diego-Garcia P, Laclaustra M. A cross-sectional study examining the Parametric Thyroid Feedback Quantile Index and its relationship with metabolic and cardiovascular diseases. Thyroid. 2022. doi: 10.1089/thy.2022.0025

Supplementary Table 2. Presence of drugs use

DRUGS	n	LOW PTFQI	COMBINED MEDIAN PTFQI: PRIMARY SUBHYPOTHYROIDISM	COMBINED MEDIAN PTFQI: MEDIAN PTFQI	COMBINED MEDIAN PTFQI: PRIMARY SUBHYPERTHYROIDISM	HIGH PTFQI	P HETER 5 groups	P TREND 3 groups
Beta-blockers	296	8.6[5]	23.9[17]	22.6[12]	32.2[19]	25.5[14]		
		1.00 (ref)	2.81(0.99, 9.33)	2.36(0.77, 8.17)	3.24(1.13,10.79)	2.13(0.71, 7.30)	0.243	0.347
Antihypertensive drugs	296	37.9[22]	47.9[34]	45.3[24]	55.9[33]	65.5[36]		
		1.00 (ref)	1.02(0.45,2.30)	0.82(0.34,1.94)	0.98(0.41,2.30)	1.46(0.60,3.54)	0.771	0.399
Lipid-lowering drugs	296	32.8[19]	52.1[37]	41.5[22]	39.0[23]	52.7[29]		
		1.00 (ref)	1.88(0.87,4.12)	1.06(0.46,2.44)	0.74(0.32,1.70)	1.28(0.56,2.95)	0.165	0.497
Antidepressants and anxiolytics	296	32.8[19]	42.3[30]	35.8[19]	39.0[23]	34.5[19]		
		1.00 (ref)	1.19(0.56,2.55)	0.93(0.41,2.09)	0.93(0.41,2.10)	0.74(0.32,1.70)	0.812	0.483

Data expressed in % [n]. OR (95% CI) from models adjusted for age and sex, referenced to the Low PTFQI group. PTFQI: Parametric Thyroid Feedback Quantile-based index. P-HETER is the p value for testing differences among groups. P-TREND is the p value from a regression model that includes group as a numerical value. “Antihipertensive drugs” includes: Angiotensin converting enzyme inhibitors, diuretics, angiotensin II receptor antagonists, mineralocorticoid antagonists. “Lipid-lowering drugs” includes: Statins, resins, cholesterol absorption inhibitors and proprotein convertase subtilisin kexin 9 enzyme inhibitors.

Supplementary Table 3. Stratified ORs for TSH and fT4 groups.

%	LEGEND		
	LOW FT4	MID FT4	HIGH FT4
HIGH TSH	PRIMARY SUBHYPOTHYROIDISM		HIGH PTFQI
MID TSH		MEDIAN PTFQI	
LOW TSH	LOW PTFQI		PRIMARY SUBHYPERTHYROIDISM

	TYPE 2 DIABETES			ISCHEMIC HEART DISEASE			ATRIAL FIBRILLATION			HYPERTENSION		
%	LOW FT4	MID FT4	HIGH FT4	LOW FT4	MID FT4	HIGH FT4	LOW FT4	MID FT4	HIGH FT4	LOW FT4	MID FT4	HIGH FT4
HIGH TSH	26.8	OR:1.55 (0.70,3.42)	41.8	8.5	OR:2.12[b] (0.71, 6.37)	16.4	9.9	OR:1.72 (0.56,5.43)	21.8	50.7	OR:1.73 (0.72,4.25)	72.7
MID TSH	OR:1.76 (0.69,4.76)	24.5	OR:1.89 (0.85,4.29)	OR:11.61[a] (0.64, 210.60)	11.3	OR:1.09[b] (0.40,2.98)	OR:6.63 (0.76,170.40)	7.5	OR:1.03 (0.39,2.75)	OR:0.83 (0.34,2.01)	52.8	OR:1.48 (0.61,3.65)
LOW TSH	13.8	OR:1.53 (0.56,4.36)	27.1	0.0	OR:22.01[a] (1.25, 387.68)	15.3	1.7	OR:8.80* (1.49,168.70)	20.3	43.1	OR:1.01 (0.38,2.59)	64.4

% of subjects with disease distributed according to PTFQI. Data expressed in OR (95% CI). ORs represent the effect of the increase in TSH or fT4 in isolation, as the other hormone does not change between compared groups. All ORs are adjusted for age and sex except where indicated.

a: unadjusted ORs calculated with Haldane-Anscombe correction. b: unadjusted ORs. *Statistically significant.

Alonso-Ventura V, Civeira F, Alvarado-Rosas A, Lou-Bonafonte JM, Calmarza P, Moreno-Franco B, Andres-Otero MJ, Calvo-Gracia F, de Diego-Garcia P, Laclaustra M. A cross-sectional study examining the Parametric Thyroid Feedback Quantile Index and its relationship with metabolic and cardiovascular diseases. Thyroid. 2022. doi: 10.1089/thy.2022.0025
